# Multilevel effective surgical workflow recognition in robotic left lateral sectionectomy with deep learning: experimental research

**DOI:** 10.1097/JS9.0000000000000559

**Published:** 2023-06-14

**Authors:** Yanzhe Liu, Shang Zhao, Gong Zhang, Xiuping Zhang, Minggen Hu, Xuan Zhang, Chenggang Li, S. Kevin Zhou, Rong Liu

**Affiliations:** a Medical School of Chinese People’s Liberation Army (PLA); Faculty of Hepato-Biliary-Pancreatic Surgery, The First Medical Center, Chinese PLA General Hospital, Beijing; b School of Biomedical Engineering & Suzhou Institute for Advanced Research, Center for Medical Imaging, Robotics, Analytic Computing & Learning (MIRACLE), University of Science and Technology of China, Suzhou, China

**Keywords:** artificial intelligence, deep learning, robotic left lateral sectionectomy, robotic surgery, surgical workflow recognition

## Abstract

**Background:**

Automated surgical workflow recognition is the foundation for computational models of medical knowledge to interpret surgical procedures. The fine-grained segmentation of the surgical process and the improvement of the accuracy of surgical workflow recognition facilitate the realization of autonomous robotic surgery. This study aimed to construct a multigranularity temporal annotation dataset of the standardized robotic left lateral sectionectomy (RLLS) and develop a deep learning-based automated model for multilevel overall and effective surgical workflow recognition.

**Methods:**

From December 2016 to May 2019, 45 cases of RLLS videos were enrolled in our dataset. All frames of RLLS videos in this study are labeled with temporal annotations. The authors defined those activities that truly contribute to the surgery as effective frames, while other activities are labeled as under-effective frames. Effective frames of all RLLS videos are annotated with three hierarchical levels of 4 steps, 12 tasks, and 26 activities. A hybrid deep learning model were used for surgical workflow recognition of steps, tasks, activities, and under-effective frames. Moreover, the authors also carried out multilevel effective surgical workflow recognition after removing under-effective frames.

**Results:**

The dataset comprises 4 383 516 annotated RLLS video frames with multilevel annotation, of which 2 418 468 frames are effective. The overall accuracies of automated recognition for Steps, Tasks, Activities, and under-effective frames are 0.82, 0.80, 0.79, and 0.85, respectively, with corresponding precision values of 0.81, 0.76, 0.60, and 0.85. In multilevel effective surgical workflow recognition, the overall accuracies were increased to 0.96, 0.88, and 0.82 for Steps, Tasks, and Activities, respectively, while the precision values were increased to 0.95, 0.80, and 0.68.

**Conclusion:**

In this study, the authors created a dataset of 45 RLLS cases with multilevel annotations and developed a hybrid deep learning model for surgical workflow recognition. The authors demonstrated a fairly higher accuracy in multilevel effective surgical workflow recognition when under-effective frames were removed. Our research could be helpful in the development of autonomous robotic surgery.

## Introduction

Highlights

- We constructed a multigranularity temporal annotation dataset of robotic left lateral sectionectomy.
- A hybrid deep learning-based automated model for multilevel surgical workflow recognition is presented.
- Our research could be helpful in the development of autonomous robotic surgery.

Since the early 2000s, experts in the field of computer-assisted surgery have been dedicated to incorporating computer technology into surgical practice^1^. By integrating systems and advancing technologies, it is expected that this will facilitate the surgical process and ultimately enhance surgical care. A clear understanding of surgical workflow is regarded as of vital importance since it helps improve operational efficiency. However, it has been a major technical problem for computers to fully comprehend surgical workflows like a surgeon due to the highly complex procedural knowledge and complicated intraoperative scenes. Over the past decade, the promising advances of artificial intelligence (AI) applications in healthcare have gradually been changing medical practice and research^2^. With the development of data science and machine learning, surgical data science (SDS) has emerged as a multidisciplinary research field, as proposed in 2016^3^. SDS may bring improvement to surgical care through the applications of context-aware assistance, decision support, and surgical training^3^. Automated surgical workflow recognition, as a representative SDS task for computer-assisted surgery, is the foundation for computational models of medical knowledge to interpret surgical procedures.

AI-based surgical workflow recognition has drawn increasing attention in minimally invasive surgery^4^. Surgical workflow recognition can benefit both surgeons and computer-assisted surgical systems, facilitating the development of context-aware system and autonomous robotic surgery. By segmenting the surgical procedure, it is possible to predict the remaining surgery duration^5^, assess the surgeon’s performance^6^, locate critical clips in surgical videos^7^, and automate certain surgical tasks^8^. Automatic workflow indexing of raw surgical videos can make it more efficient for surgeons to review the surgical process and share the essence of the surgery for education and communication^9^. Currently, researches on surgical workflow recognition have made significant strides and have achieved relatively high accuracy and precision across a range of surgical procedures^10^. However, much of this research has focused on relatively coarse-grained recognition, such as the surgical phase. Achieving autonomous robotic surgery requires more finely-grained segmentation of the surgical process, which presents a challenge for the accuracy of automated workflow recognition. Optimal surgical technique requires logical and purposeful movements, but surgeons may make unnecessary movements during the procedure. These movements not only make it difficult for surgeons to understand the surgical workflow, but they may also impact the performance of automated surgical workflow recognition. Therefore, accurate recognition of several events in a complete surgical procedure is a prerequisite for clinical applications.

Surgical temporal events at different levels indicate that a surgical video can be segmented into clips with varying granularities. Appropriate levels of granularity are critical for surgical process modeling (SPM) analysis. Notably, there are multiple categorizations for different fine-coarse descriptions of surgical procedures, such as SPM review (phase, step, activity, and motion)^11^ and the Society of American Gastrointestinal and Endoscopic Surgeons (SAGES) consensus on surgical video annotation (phase, step, task, and action)^12^. Although there are slight differences in the representation of different granularities, a hierarchical relationship between different levels of surgical events remains consistent. For example, each phase is composed of a list of steps. Through the definition of multiple hierarchies of events, researchers have greater flexibility to focus on the particular granularity level of surgical videos. However, there is a necessity for a well-defined granularity scheme since it is essential for a universal annotation framework that can generalize to various surgical datasets.

Left lateral sectionectomy (LLS) is the most commonly performed procedure among laparoscopic anatomical liver resections due to its unique anatomical characteristics and technical ease^13^. The standardized LLS procedure makes it the most suitable for surgeons learning minimally invasive hepatectomy^14,15^. Minimally invasive LLS is also a gold standard procedure, comparable to laparoscopic cholecystectomy, with more diverse instruments and a comparatively complex workflow. In our previous retrospective case-control studies, robotic and laparoscopic LLS demonstrated similar surgical workflow and perioperative outcomes^16,17^. However, compared with conventional laparoscopic surgery, robotic surgery offers advantages such as more stable vision and better ergonomics^18^. For the application of AI in surgery, the data provided by the robotic surgery platform has greater potential. The lack of standardized camera parameters and instrument types in laparoscopic surgery video data often necessitates preprocessing for AI research. However, the da Vinci robotic surgery system, with its consistent camera and precise EndoWrist instruments^19^, presents a unique opportunity for transfer learning across related surgical procedures. Unlike traditional laparoscopy, the standardized data offered by the robotic surgery system may facilitate more effective AI models for surgical video analysis. Additionally, with the hardware advantage of recording kinematic data, robotic surgery is becoming a promising platform for realizing autonomous surgery.

In this study, we constructed a multigranularity temporal annotation dataset of the standardized robotic left lateral sectionectomy (RLLS) and established a hybrid deep learning model for automated multilevel overall and effective surgical workflow recognition.

## Method

### Dataset

This study enrolls 45 patients who underwent RLLS in the Faculty of HPB Surgery, Chinese PLA General Hospital since December 2016 to May 2019. All operations were performed by four expert surgeons who had finished at least 20 RLLS previously using the da Vinci Si Surgical System (Intuitive Surgical). All the original videos were record using the Surgical Record System (Hisense Inc.) at the same frame rate (60 fps), resolution (1280×1024 pixels), and codec (MPEG-4 AVC) from the video output on the Surgeon Console of the da Vinci Si Surgical System.

The inclusion criteria are: benign or malignant lesion limited to the left lateral section (Couinaud’s segments 2 and 3) that were resected by our standardized RLLS^16,17^; liver function is Child-Pugh class A; matched surgical videos that contained complete endoscopic operations. The exclusion criteria are: a previous history of abdominal surgery requiring adhesionlysis; combined resection of other organs (such as cholecystectomy); intrahepatic cholangiocarcinoma requiring lymph node dissection; lack of corresponding surgical video, or incomplete recording of endoscopic operations.

This study was approved by the institutional ethics committees of Chinese PLA General Hospital. Written informed consent was obtained from all enrolled patients. This retrospective study was registered with ResearchRegitry.com (Unique Identification Number: researchregistry 8848). The work was reported in line with the Strengthening the Reporting of Cohort Studies in Surgery (STROCSS) criteria^20^.

### Temporal annotation

All 45 RLLS videos in this study are labeled with temporal annotations. The surgical videos in this study belong to the phase of execution of surgical objectives, as defined by the SAGES consensus^12^, and without the phase of access and closure. Therefore, in our study, step is the highest-level event rather than phase. Frames of RLLS videos were annotated with step, task, and activity based on prior studies^12,21^. The above terminology has a decreasing hierarchical relationship, representing different granularity levels of surgery. The temporal annotations in this study were made by three senior surgeons with more than 5 years of clinical experience.

Detailed surgical events of temporal annotations were discussed by expert surgeons at the Chinese PLA General Hospital. Based on previous reports from multiple medical centers and our own experiences^14,16,17,22^, we have summarized the RLLS surgical procedures with different levels in Table 1. The over-defined categories of activities with less clinical significance will make deep learning more difficult to learn useful data patterns, resulting in less satisfactory performance. To balance the domain knowledge granularity and the difficulty of training an effective deep learning model, we have only focused on the main instrument activities or effective activation of the energy device in the single frame (e.g. Harmonic Ace Curved Shears dissect the round ligament, Stapler divides the left hepatic vein, and Fenestrated Bipolar Forceps coagulate liver parenchyma). Note that simultaneous activities performed by auxiliary instruments are not necessary to be annotated since the fine-level information would not help too much in exploring the importance of filtering behaviors during idle time, such as fenestrated bipolar forceps grasp round ligament, fenestrated bipolar forceps retract liver, and irrigator aspirates fluid.

**Table 1:** Multilevel temporal annotations of RLLS.

Step	Task	Activity
S1 (Mobilization of the liver)	T1 (Dissection of the round ligament)	AA1 (Shears^*^ dissect round ligament) AB1 (Hook^*^ dissects round ligament) AC1 (Forceps^*^ coagulate round ligament).
	T2 (Dissection of the falciform ligament)	AA2 (Shears dissect falciform ligament) AB2 (Hook dissects falciform ligament).
	T3 (Dissection of the left coronary ligament)	AA3 (Shears dissect left coronary ligament) AB3 (Hook dissects left coronary ligament) AC2 (Forceps coagulate left coronary ligament).
	T4 (Dissection of the left triangle ligament)	AA4 (Shears dissect left triangle ligament) AB4 (Hook dissects left triangle ligament) AC3 (Forceps coagulate left triangle ligament).
	T5 (Dissection of the hepatogastric ligament)	AA5 (Shears dissect hepatogastric ligament) AB5 (Hook dissects hepatogastric ligament) AC4 (Forceps coagulate hepatogastric ligament).
S2 (Parenchymal transection)	T6 (Transection of the liver parenchyma)	AA6 (Shears dissect liver parenchyma) AC5 (Forceps coagulate liver parenchyma) AD2 (Stapler divides liver parenchyma) AI1 (Clipper clip vessels).
	T7 (Division of the Glissonian pedicles of Segments 2 and 3)	AD1 (Stapler divides glissonian pedicles of Segments 2 and 3).
	T8 (Division of the left hepatic vein)	AD3 (Stapler divides left hepatic vein) AI1 (Clipper clip vessels)
S3 (Hemostasis after transection)	T9 (Hemostasis of the transected liver surface)	AB6 (Hook coagulates liver parenchyma) AC5 (Forceps coagulates liver parenchyma) AF1 (Needle sutures liver parenchyma) AG1 (Coagulator^*^ coagulates liver parenchyma) AH1 (BiClamp coagulates liver parenchyma).
	T10 (Hemostasis of the perihepatic tissues)	AC1 (Forceps coagulate round ligament) AC4 (Forceps coagulate hepatogastric ligament).
S4 (Extraction and drainage)	T11 (Package of the specimen)	AE1 (Endobag packs resected specimen).
	T12 (Drainage tubes placement)	AJ1 (Drainage tube places in resected liver Bed).

* Shears: Harmonic Ace Curved Shears, Hook: Permanent Cautery Hook, Forceps: Fenestrated Bipolar Forceps, Coagulator: Argon Beam Coagulator.

**Figure 1:**
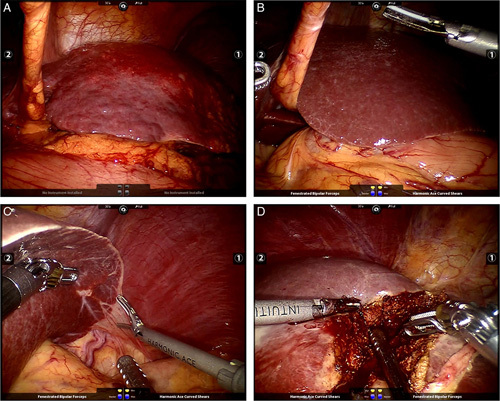
Illustrative images of under-effective frames. (A) Instrument-free state. (B) Nonvisualized activities. (C) Preparation state before activation. (D) Activities of auxiliary instruments.

Idle time is defined as the absence of any action visualized in the endoscopic view^12^. Moreover, the less meaningful context in idle frames will cost extra efforts of the deep learning models in distinguishing the significant patterns and noisy idle patterns for workflow understanding. Therefore, its presence can undoubtedly affect the accuracy of surgical workflow recognition and can be excluded when necessary. This is similar to the logic of surgical video editing for surgery education. To utilize the deep learning method to analyze the impact of idle time on the efficiency of surgical workflow recognition using deep learning, we have introduced the terms ‘effective’ and ‘under-effective’ into surgical temporal annotation. Effective frames are defined as those in which the surgical instrument is acting on the tissue in an activated state, representing a series of activities that truly contribute to the surgery. Under-effective frames refer to surgical activities other than effective frames, such as instrument-free state, nonvisualized activities (i.e. idle time), preparation state before activation, and activities of auxiliary instruments (Fig. 1).

Step refers to specific parts of a surgery that are necessary to accomplish a clinically meaningful goal, such as mobilization of the liver. In most cases, steps are carried out in a specific order, but sometimes they can be interrupted. Additionally, a step is composed of a sequence of tasks. A task is the lower-level surgical temporal event required to achieve an objective. For instance, the mobilization of the liver requires the tasks of dissecting perihepatic ligaments (e.g. the round ligament and the falciform ligament). In the annotation at the activity level, we use activity triplets to represent the instrument used, the action performed, and the object acted upon^23^. For example, the statement ‘the Harmonic Ace Curved Shears (Shears) dissects the round ligament’ is expressed as (Shears, Dissect, Round ligament). The introduction of activity triplets is useful for further analysis of instrument-action-object interactions.

**Figure 2:**
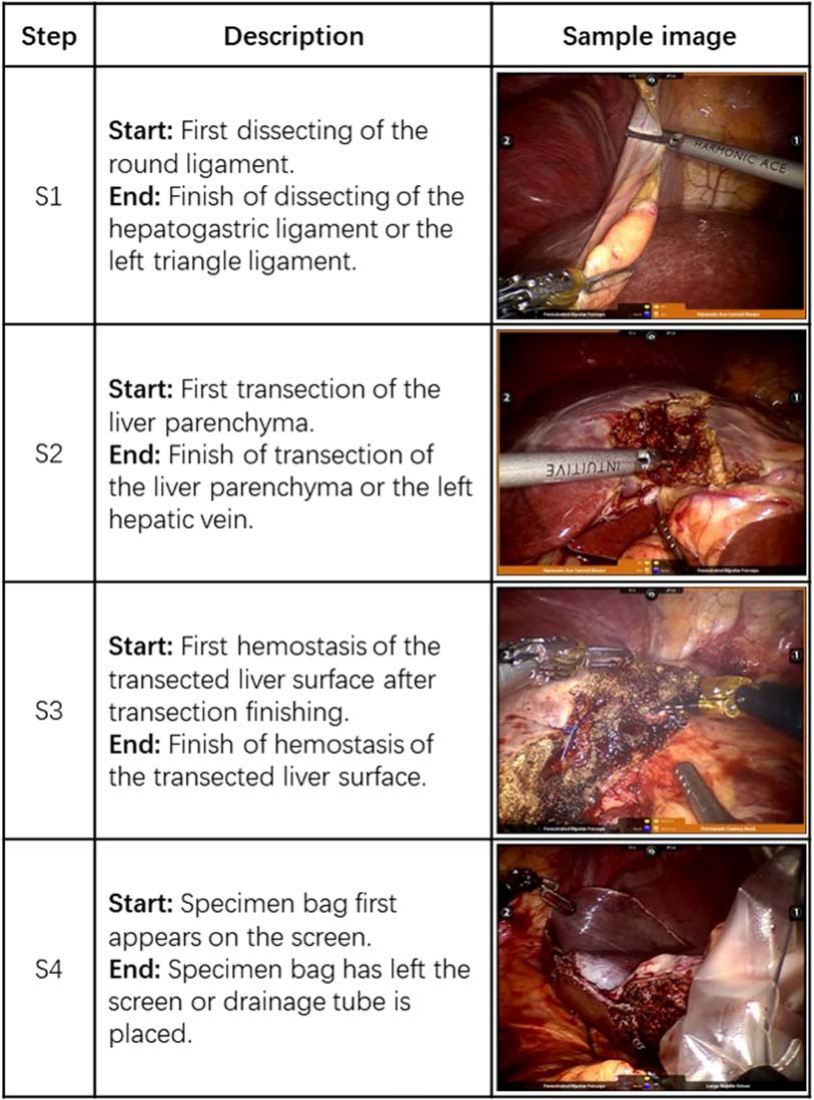
Description and sample images of Step in RLLS.

**Figure 3:**
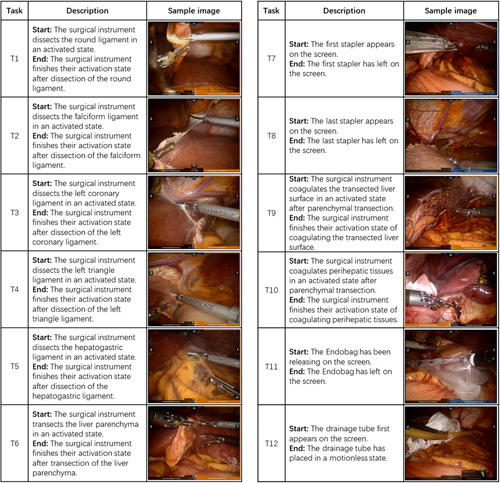
Description and sample images of Task in RLLS.

**Figure 4:**
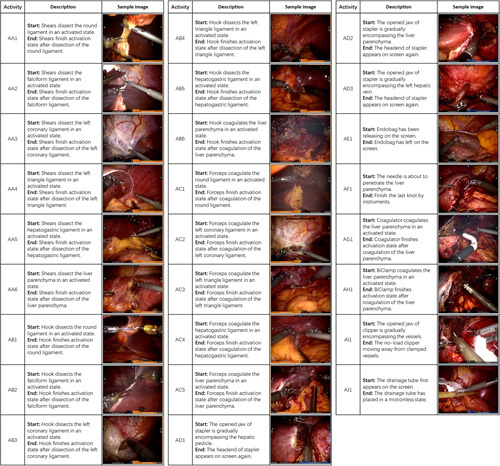
Description and sample images of Activity in RLLS.

We present a summary of the starting and ending points for each Step, Task, and Activity in Figures 2, 3, and 4, respectively. A Step typically comprises several fixed Tasks; for example, the mobilization of the liver (S1) consists of T1–T5. To define a complete period of a Step, we use the beginning of its first Task as the start and the finishing of the last Task in the Step as the end. Similarly, we apply the same principle on defining the relationship between Tasks and Activities. We begin our temporal annotation at the most concrete description level, which is the Activity, annotating the starting point when the instrument is effectively acting on the object, and annotating the out-point when the Activity is complete. When the Activity annotation for a temporal fragment is complete, the corresponding higher-level Tasks and Steps can also be assigned. Following the aforementioned rules, we can efficiently annotate valid surgical temporal fragments, and consider the rest frames as under-effective ones. Specifically, when the same activity has an interval of less than 10 s, we still consider it as a continuous effective temporal fragment. Otherwise, we consider it an under-effective fragment.

### Multilevel surgical workflow recognition

**Figure 5:**
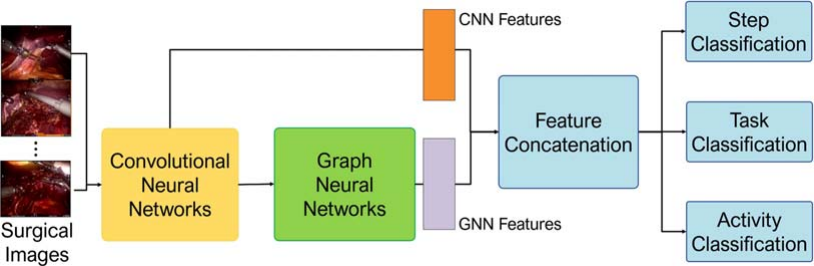
The hybrid deep learning model for multilevel surgical workflow recognition.

In this study, we utilize a hybrid deep learning model which comprises a convolutional neural network (CNN) module and a graph neural network (GNN) module to jointly learn useful information from endoscopic images, as shown in Figure 5. The CNN encoder module first extracts low-level structural context in a hierarchical fashion, then the high-level context features are further polished through the GNN module. We use a graph convolutional operator to aggregate the frame features based on the metric of cosine CNN feature correlation by constituting an affinity matrix. The affinity information describes the neighboring information for the graph convolutional operation to integrate the neighboring CNN feature information of annotated frames in batches, where the neighbor for each sample is defined as the frames in the batch which has the same annotation. The aggregated features then pass to the next graph convolutional layer for further processing. After having both features, we apply a fusion module to concatenate both types of features for surgical behavior identification. The CNN feature emphasizes more on the spatial features of images, and the GNN feature focuses more on the descriptions of spatial feature similarity between images. In this way, our model utilizes hybrid features, which are CNN and GNN features, to comprehensively describe the useful information from different perspectives. Each downstream task comprises a fully connected layer for classification, the output dimension is the number of classes in the annotation type. We carefully set the importance of different classification tasks to encourage the network to emphasize more the complex fine-level tasks such as Activity recognition.

In our training configuration, we split the dataset into training and testing sets by experts. In detail, we use experts B and C for training and experts A and D for testing, which is in a 34:11 split. This design makes the partition maintain the independence of surgical styles. Following the related works^24^, we sample the videos into frames with a fixed 5fps sampling rate, and then resize the endoscopic images to a resolution of 256×320. Color image augmentation such as color balancing and the random crop is also optional to use for increasing the robustness of feature representation. The CNN module is a ResNet50 initialized with the pretrain parameters on the ImageNet. The GNN module is a 2-layered GNN module^25^. Specifically, we use stochastic gradient descent optimizer for training by setting the learning rate to 0.001. The learning rate is tuned with an exponential learning rate scheduler, the learning rate decay rate is set to 0.99. The batch size is set to 16. Each model is trained with 50 epochs and we utilize the result of the last epoch for comparison.

We design two types of experiments for evaluating the importance of filtering under-effective frames. To realize the objective, the first configuration is defined as learning all types of annotations including under-effective frames. The other is a progressive design that first filters out the under-effective frames and then recognizes the valid multileveled surgical behaviors with our hybrid networks.

To evaluate the effectiveness of the progressive approach, we report the model performance with the following metrics: accuracy, precision, recall, and F-1 scores, and their definitions are listed below, TP, FP, TN, and FN represent true positive, false positive, true negative, and false negative, respectively. The formulations of the evaluation metrics are listed below:


$$\mathrm{Accuracy}=\frac{\mathrm{TP}+\mathrm{TN}}{\mathrm{TP}+\mathrm{FP}+\mathrm{TN}+\mathrm{FN}}$$

$$\mathrm{Precision}=\frac{\mathrm{TP}}{\mathrm{TP}+\mathrm{FP}}$$

$$\mathrm{Recall}=\frac{\mathrm{TP}}{\mathrm{TP}+\mathrm{FN}}$$

$$F1\;\mathrm{score}=2\times \frac{\mathrm{Pr}\mathrm{ecision}\times \mathrm{Recall}}{\mathrm{Precision}+\mathrm{Recall}}$$

## Result

### Patient characteristics

Of the 45 RLLS patients who were included into this study, there was 21 males and 24 females (Table 2). The average age was 53.53±14.70 years old. The average BMI was 23.91±3.71, and the average lesion diameter was 5.45±2.93 cm. The average operation time was 77.82±22.28 min, and the average blood loss was 45.89±72.61 ml. There were 19 patients with primary hepatocellular carcinoma, 15 patients with hemangioma, 5 patients with focal nodular hyperplasia, 2 patients with biliary cystadenoma, 1 patient with colorectal liver metastases, 1 patient with epithelioid angiomyolipoma, 1 patient with a solitary necrotic nodule, and 1 patient with tuberculosis.

**Table 2:** Patient characteristics and pathology.

Characteristic	RLLS (*n*=45)
Age (years)	53.53±14.70
Sex (male/female)	21/24
BMI (kg/m^2^)	23.91±3.71
Lesion diameter (cm)	5.45±2.93
Cirrhosis	11 (24.4%)
Previous abdominal surgery	8 (17.8%)
Pathology	
Biliary cystadenoma	2 (4.4%)
Colorectal liver metastases	1 (2.2%)
Epithelioid angiomyolipoma	1 (2.2%)
Focal nodular hyperplasia	5 (11.1%)
Hemangioma	15 (33.3%)
Hepatocellular carcinoma	19 (42.2%)
Solitary necrotizing nodule	1 (2.2%)
Tuberculosis	1 (2.2%)

### RLLS dataset

**Figure 6:**
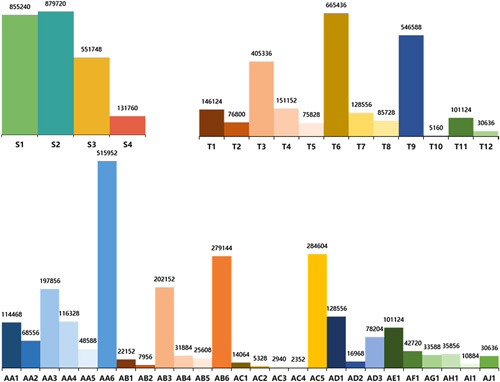
The distribution of Step, Task, and Activity annotations.

The dataset comprises 4 383 516 annotated RLLS video frames with hierarchical annotation, of which 2 418 468 frames are effective. Figure 6 shows the distribution of Step, Task, and Activity annotations. Among the various Step events, liver parenchyma transection (S2) has the highest proportion, accounting for 879 720 frames. Correspondingly, transection of the liver parenchyma (T6) has the largest number of events in the Task category, followed by the hemostasis of transected liver surface (T9). In the Activity statistics, the top five events are AA6, AC6, AB7, AA3, and AD1.

### Surgical workflow recognition

**Figure 7:**
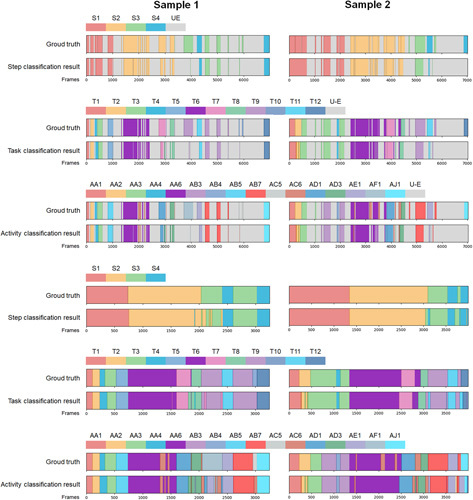
Multilevel surgical workflow recognition results of two RLLS example.

We present the multilevel surgical workflow recognition results of two RLLS example in Figure 7, while the overall surgical workflow recognition results are shown in Table 3. The overall accuracies of automated recognition for Steps, Tasks, Activities, and Under-effective frames are 0.82, 0.80, 0.79, and 0.85, respectively, with corresponding precision values of 0.81, 0.76, 0.60, and 0.85. We also conducted effective surgical workflow recognition after removing under-effective frames, resulting in a significant improvement in performance compared to the overall surgical workflow recognition. The overall accuracies were increased to 0.96, 0.88, and 0.82 for Steps, Tasks, and Activities, respectively, while the precision values were increased to 0.95, 0.80, and 0.68 (Table 4).

**Table 3:** Accuracy, Precision, Recall and F1-scores for overall automated surgical workflow recognition.

	Accuracy	Precision	Recall	F1-scores
Step	0.82	0.81	0.77	0.79
Task	0.80	0.76	0.60	0.65
Activity	0.79	0.60	0.44	0.47
Under-effective	0.85	0.85	0.85	0.85

**Table 4:** Accuracy, precision, recall and F1-scores for effective automated surgical workflow recognition.

	Accuracy	Precision	Recall	F1-scores
Step	0.96	0.95	0.95	0.95
Task	0.88	0.80	0.78	0.78
Activity	0.82	0.68	0.58	0.60

The effective surgical workflow recognition efficiency of the deep learning network for each event in Step, Task and Activity are shown in Table 5. The average precision for Step is 0.97±0.01. For Task, the average precision is 0.84±0.19, with T10 having the lowest precision of only 0.32. The average accuracy of Activity is 0.82±0.21, with AC4, AD2, and AC1 having the lowest precision values of 0, 0.50, and 0.56, respectively.

**Table 5:** Precision, recall, and F1-scores of each event in effective automated surgical workflow recognition.

	Precision	Recall	F1-scores	Frames
S1	0.98	0.97	0.98	855 240
S2	0.97	0.97	0.97	879 720
S3	0.97	0.96	0.97	551 748
S4	0.94	0.96	0.95	131 760
T1	0.96	0.95	0.95	146 124
T2	0.87	0.89	0.88	76 800
T3	0.94	0.87	0.90	405 336
T4	0.79	0.92	0.85	151 152
T5	0.90	0.82	0.86	75 828
T6	0.95	0.94	0.95	665 436
T7	0.84	0.75	0.79	128 556
T8	0.65	0.81	0.72	85 728
T9	0.96	0.97	0.96	546 588
T10	0.32	0.14	0.20	5160
T11	0.90	0.96	0.93	101 124
T12	0.97	0.84	0.90	30 636
AA1	0.95	0.96	0.95	114 468
AA2	0.86	0.88	0.87	68 556
AA3	0.93	0.75	0.83	197 856
AA4	0.77	0.95	0.85	116 328
AA5	0.90	0.80	0.84	48 588
AA6	0.92	0.94	0.93	515 952
AB1	0.96	0.93	0.94	22 152
AB2	0.87	0.86	0.86	7956
AB3	0.84	0.98	0.91	202 152
AB4	0.80	0.85	0.82	31 884
AB5	0.98	0.80	0.88	25 608
AB6	0.94	0.97	0.96	279 144
AC1	0.56	0.47	0.51	14 064
AC2	0.87	0.33	0.48	5328
AC3	1.00	0.16	0.28	2940
AC4	0.00	0.00	0.00	2352
AC5	0.81	0.86	0.83	284 604
AD1	0.82	0.79	0.80	128 556
AD2	0.50	0.22	0.31	16 968
AD3	0.62	0.80	0.70	78 204
AE1	0.88	0.97	0.92	101 124
AF1	0.99	0.80	0.88	42 720
AG1	0.81	0.47	0.60	33 588
AH1	0.95	0.86	0.90	35 856
AI1	0.78	0.58	0.66	10 884
AJ1	0.95	0.84	0.89	30 636

## Discussion

In this study, we developed a multilevel dataset comprising 45 cases of RLLS and employed deep learning to automatically recognize an overall and effective surgical workflow. The recognition of Step, Task, Activity, and Under-effective was performed using a CNN+GNN model. In the overall surgical workflow recognition, the accuracies of Step, Task, and Activity were 0.82, 0.80, and 0.79, respectively. Notably, the effective surgical workflow recognition without under-effective frames achieved higher accuracies of 0.96, 0.88, and 0.82. The existence of under-effective frames not only degrades the performance of model recognition, but also reduces the efficiency of surgeons in learning surgery. During clinical procedures, surgeons often engage in repetitive and inefficient manipulations that can extend the duration of an operation by several hours. However, showcasing the entirety of each surgery can be a time-consuming endeavor. As a result, surgeons frequently edit out under-effective frames and highlight key sections of surgical videos that are used for teaching and presentations. Our findings demonstrate that removing under-effective frames can improve the performance of the deep learning model for multilevel surgical workflow recognition, which is beneficial for further surgical navigation and cognitive systems in autonomous robotic surgery. To the best of our knowledge, this is the first study to employ a deep learning model to improve understanding of surgical scenarios by using multilevel effective surgical workflow recognition in RLLS.

Surgical workflow recognition is a foundational task and an important research area for AI applications in surgery. Traditional machine learning models, such as dynamic time warping, hidden Markov model, and support vector machine, were the primary tools used for surgical workflow recognition in the early stage. However, deep learning model have been most popularly for automated surgical workflow recognition now^4,26^. Compared to conventional machine learning models, deep learning models offer several advantages in terms of image data feature extraction and learning, albeit with higher requirements for data labeling and computational power. In our study, we opted for a hybrid deep learning model combining CNN+GNN, due to the complex task requirements and the availability of sufficient labeled data. Laparoscopic cholecystectomy is the most commonly used procedure to test automated surgical workflow recognition models^4^. Other procedures that have been used for this task include liver surgery^27^, gynecologic surgery^28^, colorectal surgery^29^, bariatric surgery^30^, and urological surgery^31^. In our study, owing to the standardized process of RLLS, multilevel surgical workflow recognition achieved relatively high accuracy. Different surgical procedures in liver surgery share similar surgical steps and tasks, while finer-grained actions are more specific to each procedure. For instance, both left hemi-hepatectomy and LLS share steps such as mobilization of the liver, parenchymal transection, hemostasis after transection, extraction, and drainage, while differing slightly in finer-grained tasks and activities. Therefore, the models trained in this study can be used for transfer learning in further research.

Most surgical workflow recognition studies have focused on coarse-grained level recognition, such as phase recognition. However, standardization of ontologies at different granularities in surgical workflow remains a significant challenge. For example, in workflow recognition studies focused on LC, the number of phases varies from 6 to 20^32–35^. To address this issue, the OntoSPM project and the SAGES consensus aim to define consistent ontologies in surgical video annotation^12,36^. Some researchers have used multitask and multistage neural networks to jointly recognize the phases and steps of laparoscopic gastric bypass surgeries^37^. In robotic surgery research, kinematic data and finer-grained annotation have enabled researchers to focus on recognizing gestures associated with specific surgical activities^38–40^. In our study, we segmented each RLLS procedure into a multileveled interpretation, including step, task, and activity, enabling surgical workflow analysis at different levels from high to low. The automatic recognition of step and task are beneficial to the catalog management of surgical videos, and facilitates retrospective analysis, surgical communication and learning for surgeons and researchers. In addition, the automatic recognition of activity is more conducive to motion analysis in autonomous robotic surgery.

Activity, as a fine-grained temporal event defined in this study, can be used to directly represent a concrete surgical status. It captures the surgical interaction between instruments and tissue, and is often described as a triplet of the instruments used, the action performed, and the object acted upon^21^. Activity triplets are an expressive form for surgical workflow recognition^23^, as they provide efficient details for recognizing different levels of granularity. Thus, the recognition of surgical steps and tasks can be based on the recognition of activity triplets. We achieved high precision in recognizing most activities, but some cases showed poor recognition, such as ‘forceps coagulate hepatogastric ligament (AC4)’ and ‘forceps coagulate round ligament (AC1)’, which had precision scores of only 0 and 0.56, respectively. This was mainly due to the deficiency and maldistribution of data, which is a representative challenging technical problem in practical deep learning. Moreover, we found that the accuracy of recognition improved as the number of training frames increased, further confirming our assumption. In addition, activity information contains the description of instrument and tissues, which is useful for finer-grained motion modeling and gesture segmentation^41^. In surgical image analysis, temporal annotation of instruments and tissue can provide weakly supervised labels for classifying and detecting surgical instruments and anatomical structures. However, under-effective frames can interfere with the surgical workflow recognition of deep learning models. Our results show that removing such frames significantly improves the performance of recognition.

With the rapid development of AI technology, the autonomy levels of surgical robotics are expected to continue to increase^42^. For autonomous surgical robotics to be effective, they must be able to perceive the surgical environment and perform surgical actions with precision^43^. Similar to the training of a novice surgeon, surgical robotics must also be taught how to perform surgery. A fundamental step towards achieving this goal is to segment an entire surgical procedure into episodes^44^. Surgical videos are a valuable data source for deep learning algorithms used in autonomous robotic surgery, facilitating surgical workflow analysis and surgical image analysis. This study demonstrates that in an idealized surgical operation setting, deep learning models perform better in understanding the surgical workflow. The recognition of under-effective frames can facilitate the deep learning model in future automatic editing of surgical videos. Additionally, the activity triplet form of instrument-action-object in this study can be integrated with surgical image analysis to improve the deep learning understanding of surgery. Finally, the integration of AI technology into surgical practice has brought a series of ethical concerns^3,42,45^. To safeguard patient confidentiality, the security and privacy of clinical data must be prioritized during data acquisition and storage. Furthermore, the potential for algorithmic bias to be introduced during model training calls for the implementation of debiasing and counterfactual analysis techniques to enhance model transparency and repeatability. A comprehensive ethical framework must be established to guide the development and deployment of AI systems in surgery, taking into account the unique challenges and risks of this rapidly evolving field.

The present study has several limitations that must be acknowledged. First, the cases included in this study were acquired from a single center, which may lead to overfitting of the deep learning model. Although robotic anatomical hepatectomy is a classic procedure, some variations exist between different institutions. For instance, while the Pringle maneuver is commonly used for hepatic inflow occlusion before transection of the liver parenchyma, none of the 45 cases in our study utilized this technique. Second, although we achieved a fairly good performance by removing under-effective frames, the recognition rate of some tasks and activities remains inadequate for practical application. This inadequacy is mostly due to insufficient training data, such as Hemostasis of the perihepatic tissues (T10) in Task, AC1 and AC4 in Activity. Furthermore, we observed that some inaccuracies were caused by small differences in the videos, such as the Stapler divides liver parenchyma (AD2) and Stapler divides left hepatic vein (AD3), with accuracies of only 0.5 and 0.62, respectively. Therefore, future research should focus on increasing the training sample size to improve the model’s accuracy. As data volume increases and data sources become more diverse, the deep learning model’s robustness will also be enhanced. Finally, the lack of kinematic data of surgical instruments in this study may limit further research on autonomous surgery. The current public datasets mainly include kinematic data from the da Vinci Research Kit, the research version of the Da Vinci for in-vivo and ex-vivo experiments. Therefore, future research may need to incorporate images and kinematic data from existing datasets, such as JIGSAW^41^ and UCL da Vinci Research Kit^46^, to achieve a deeper level of motion segmentation.

## Conclusion

In this study, we created a dataset of 45 RLLS cases with multilevel annotations and developed a hybrid deep learning model for surgical workflow recognition. Our model achieved high accuracy in recognizing steps, tasks, and activities on this dataset. Furthermore, we demonstrated a fairly high accuracy in multilevel effective surgical workflow recognition when under-effective frames were removed. Our research contributes to the understanding of the surgical workflow by deep learning models, which could be helpful to the development of autonomous robotic surgery.

## Ethical approval and consent to participate

This study was approved by the Institutional Review Board of the Chinses People’s Liberation Army General Hospital (S2016-098-02). Written informed consent was obtained from all enrolled patients.

## Consent for publication

Consent for publication was obtained from all authors.

## Sources of funding

This work was supported by the National Key R&D Program of China (Grant 2021ZD0113301).

## Author contribution

R.L., S.K.Z., Y.L., S.Z.: conception and design; R.L., S.K.Z.: financial support; R.L., M.H., X.Z., C.L.: provision of study materials or patients; Y.L., G.Z., X.Z.: collection and assembly of data; Y.L., S.Z.: data analysis and interpretation; Y.L., S.Z., G.Z., X.Z.: manuscript writing; Final approval of manuscript is done by all authors.

## Conflicts of interest disclosure

No potential conflicts of interest were disclosed.

## Research registration unique identifying number (UIN)

The study was registered with ResearchRegistry.com. Research Registration Unique Identifying Number is researchregistry8848.

## Guarantor

Rong Liu, S. Kevin Zhou.

## Availability of data and materials

Data sharing is not applicable to this article.
